# Milk-Alkali Syndrome in the Context of Pulmonary Tuberculosis: An Overlooked Aetiology of Hypercalcaemia

**DOI:** 10.7759/cureus.96369

**Published:** 2025-11-08

**Authors:** Ali Bani-Mustafa, Ahmed Hegazy, Kashif Khan, Mohammed Saad

**Affiliations:** 1 Respiratory Medicine, Medway NHS Foundation Trust, Gillingham, GBR; 2 Acute Medicine, Medway Maritime Hospital, Gillingham, GBR; 3 Acute Medicine, General Internal Medicine, Medway NHS Foundation Trust, Gillingham, GBR

**Keywords:** calcium intake, hypercalcemia, metabolic alkalosis, milk-alkali syndrome, pulmonary tuberculosis

## Abstract

A 64-year-old male with a history of alcoholism and newly diagnosed pulmonary tuberculosis (TB) developed hypercalcaemia during anti-TB treatment (Rifampicin, Isoniazid, Pyrazinamide, and Ethambutol). He manifested right leg bone pain and hallucinations. Hypercalcaemia workup, including parathyroid hormone (PTH), vitamin D, myeloma screen, and imaging, revealed no underlying malignancy or granulomatous bone involvement. Despite stopping vitamin D/calcium supplements and receiving fluids, bisphosphonates, calcitonin, and steroids, his calcium levels remained elevated. Further history revealed excessive milk intake (1-2 L/day). Cessation of dairy led to gradual symptom resolution and normalisation of serum calcium within two weeks. This case highlights milk-alkali syndrome as a rare but reversible cause of hypercalcaemia in TB patients.

## Introduction

In 1915, Sippy introduced an antacid regimen aimed at neutralising gastric acid and promoting the healing of peptic ulcer disease [1]. This treatment involved the hourly intake of milk or cream combined with powders containing magnesium carbonate and sodium bicarbonate, alternating with powders composed of bismuth subcarbonate and sodium bicarbonate. Although toxic effects such as alkalosis and kidney injury were soon reported, plasma calcium levels were not measured at that time [2]. It was not until 1936 that a report linked hypercalcaemia to the alkalosis and renal damage seen in patients receiving the Sippy regimen [3]. With the advent of modern histamine (H2) receptor antagonists and proton pump inhibitors (PPIs) for peptic ulcer disease, this condition has nearly vanished [4]. While the theory behind the use of large amounts of milk may seem like a relic of the past, rarely encountered in the era of modern acid-suppressive therapies, our case today serves as a reminder that the effects of excessive milk consumption - and its potential consequences - still persist in clinical practice. With this in mind, we present the following case that takes us back to the early days of Bertram Sippy’s regimen.

## Case presentation

A 64-year-old male with no significant past medical history, apart from alcoholism, was admitted to the respiratory unit for treatment of newly diagnosed pulmonary tuberculosis (TB). He was receiving anti-TB medications, including Rifampicin, Isoniazid, Pyrazinamide, and Ethambutol, when he developed hypercalcaemia. At that time, he complained of bone pain in his right leg and reported experiencing hallucinations.

A hypercalcaemia workup, including parathyroid hormone (PTH), vitamin D, alkaline phosphatase, and a multiple myeloma screen (Table 1), was conducted but did not reveal any underlying cause.

**Table 1:** Serial laboratory values during admission and treatment of hypercalcaemia.

	Admission	First day of hypercalcaemia (Day 1)	Day 8	Milk stop day (Day 14)	Day 21	Day 28	On discharge (Day 49)	Reference range	
									Hb (g/L)	147	132	122	145	120	113	108	130-170	
WBC (×10^9^/L)	5.6	14.2	7.6	6.8	10	9.5	7.8	04-11	
Platelets (×10^9^/L)	405	605	427	336	270	274	333	150-400	
Adjusted calcium (mmol/L)	2.18	2.86	3.38	3.29	2.79	2.52	2.46	2.20-2.60	
Phosphate (mmol/L)	0.94	1.5	1	1	1	1.22	1.44	0.80-1.50	
Alkaline phosphatase (U/L)		103	75	87	100	69	57	30-130	
Albumin (g/L)	30	27	22	29	28	32	33	35-50	
Vitamin D (nmol/L)	16	-	-	-	-	-	-	>50	
PTH (ng/L)		<1	3	-	-	-	-	12-88	
IgG (g/L)	8.2	-	-	-	-	-	-	7.0-16.0	
IgA (g/L)	2.48	-	-	-	-	-	-	0.7-4.0	
IgM (g/L)	0.83	-	-	-	-	-	-	0.4-2.3	
GFR (mL/min/1.73 m^2^)	>90	>90	>90	>90	>90	>90	>90	>90	
Creatinine (μmol/L)	70	55	51	67	66	53	52	64-104	
Na (mmol/L)	131	136	141	140	139	140	138	136-145	
K (mmol/L)	4.9	3.9	3.4	3.4	3.5	4.2	4.6	3.5 - 5	
Urea (mmol/L)	6.6	4.5	5.5	4.8	7.3	11.3	8.2	2.5-7.8	
Mg (mmol/L)	0.9	0.71	0.71	0.77	0.79	0.77	0.8	0.7-1.0	
CRP (mg/L)	167	121	39.4	20	6.7	14.4	13.8	0.0-5.0	
PH	7.48	-	-	7.53	-	-	-	7.32-7.43	
pCO2 (kPa)	4.6	-	-	5.4	-	-	-	4.6-6	
HCO3 (mmol/L)	26.5	-	-	32.5	-	-	-	22-29	

**Figure 1:**
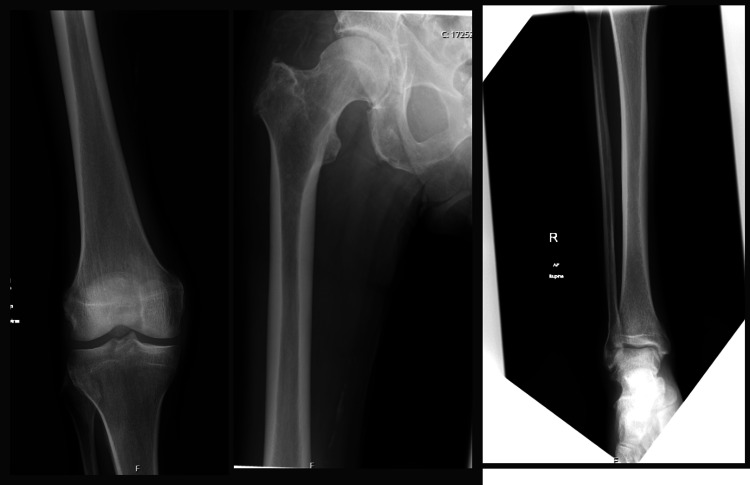
X-ray femur, tibia, and fibula showing mild degenerative changes with no osteolytic lesions.

**Figure 2:**
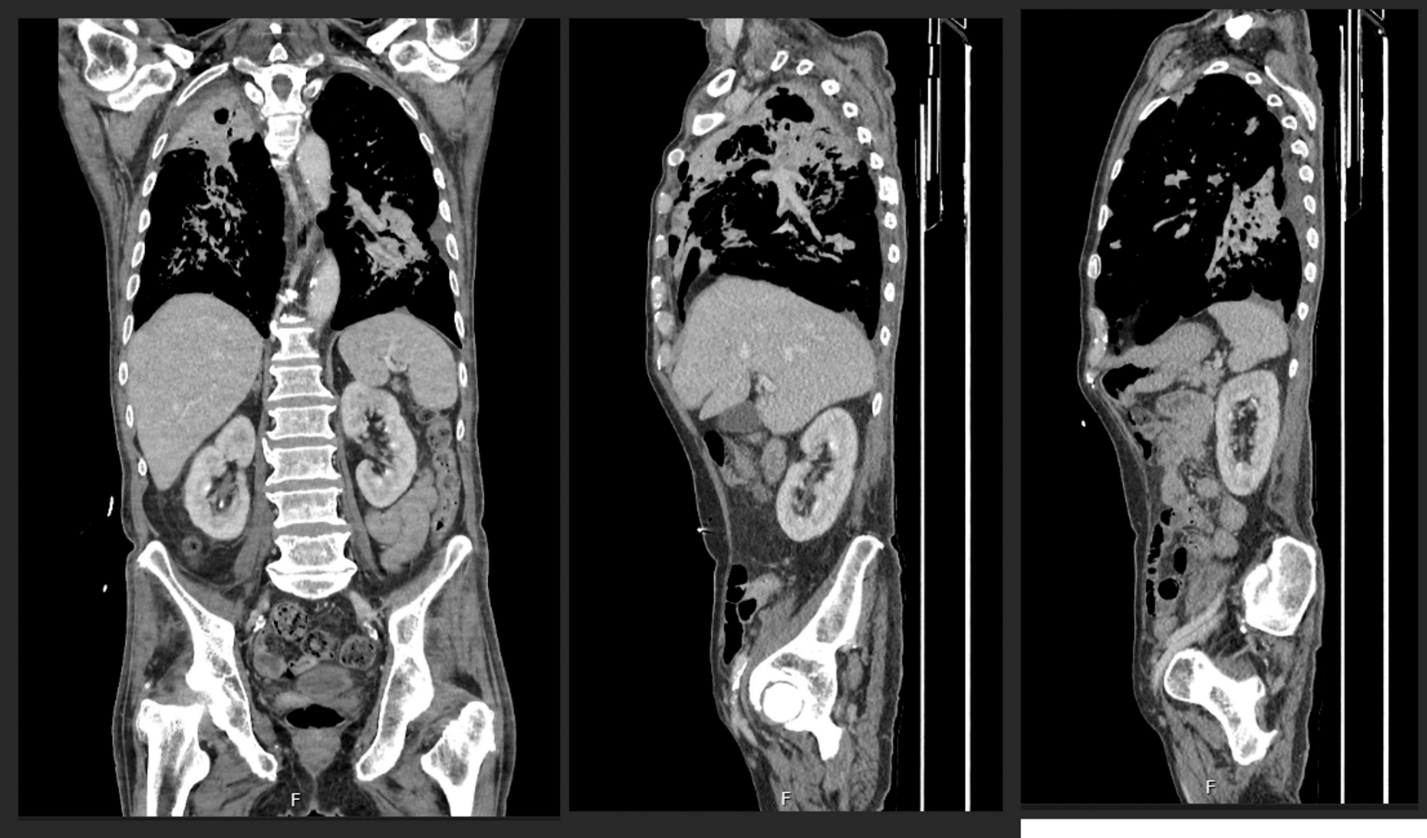
CT chest, abdomen, and pelvis showing stigmata of pulmonary TB and no signs of malignancy.

**Figure 3:**
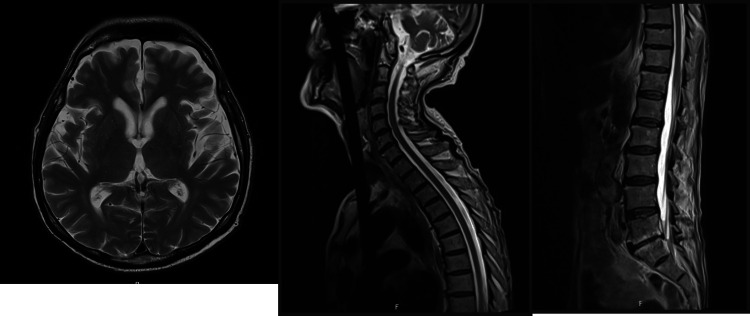
Normal MRI head and whole spine.

X-rays of the right femur, tibia, and fibula revealed only mild degenerative changes (Figure 1). CT scans of the head, neck, chest, abdomen, and pelvis (Figure 2), together with MRI scans of the head and entire spine (Figure 3), showed no evidence of malignancy or osteolytic lesions. Furthermore, the aforementioned imaging also excluded the presence of osteoporosis, osteoarthritis, and fractures. 

Arterial blood gas (ABG) analysis revealed metabolic alkalosis, while his kidney function remained unaffected (Table 1).

The patient was taking vitamin D and calcium supplements for vitamin D deficiency, which were withheld upon the discovery of hypercalcaemia, but there was no change in his serum calcium levels. He was not on any other medications known to cause hypercalcaemia.

Several treatments were attempted, including liberal intravenous crystalloids, bisphosphonates, calcitonin, and glucocorticoids, as it was suspected that the hypercalcaemia might be secondary to his granulomatous disease (TB). However, these interventions did not lead to a significant reduction in his serum calcium level.

**Figure 4:**
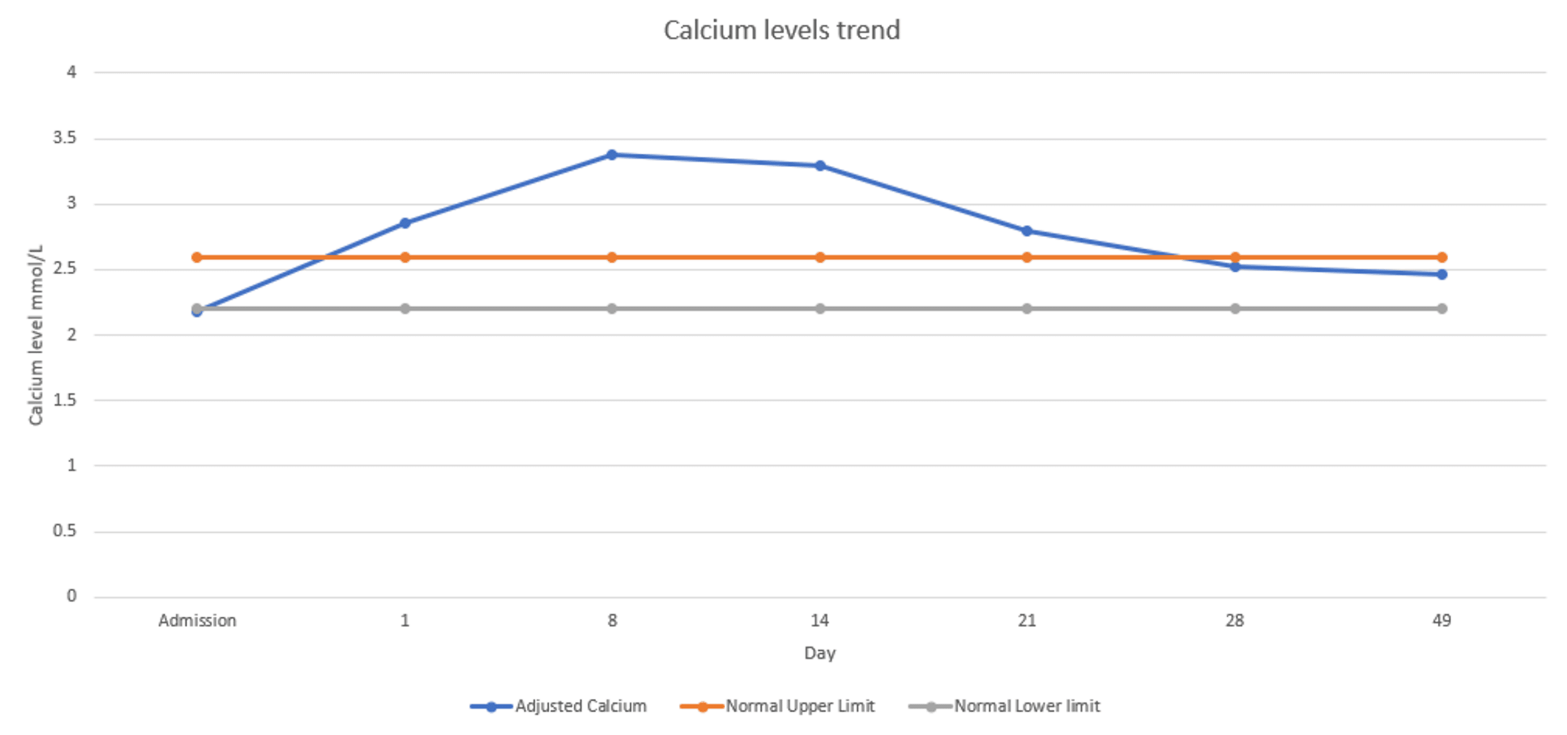
Calcium levels trend.

It was then discovered that he was consuming large quantities of milk daily, 1-2 L, which contains about 1.5-2.6 g of calcium. He was advised to stop consuming dairy products. A week later, his symptoms improved gradually, his serum calcium levels began to decrease, and they normalised after another week (Figure 4).

## Discussion

Milk-alkali syndrome (MAS) is a rare but important cause of hypercalcaemia, historically associated with the excessive intake of milk and absorbable alkali, most notably calcium carbonate. Fiorino investigated this syndrome by randomly assigning 40 men, most with a history of peptic ulcer disease, to receive 6 oz of milk and either 3.6 g of calcium carbonate or 15 ml of aluminium hydroxide or aluminium magnesium hydroxide every two hours while awake-equating to daily averages of approximately 1.5 L of milk and 28 g of calcium carbonate in the calcium group [5]. They found significant increases in serum calcium, phosphate, creatinine, and bicarbonate in the calcium carbonate group compared to the aluminium-based antacid group, as well as elevated urinary calcium excretion. One subject developed full clinical features of the syndrome, including marked elevations in serum calcium, phosphate, urea nitrogen, creatinine, and bicarbonate, all of which normalised following cessation of milk and calcium carbonate intake.

In our case, calcium and vitamin D supplementation had already been discontinued one week prior to the patient reaching peak serum calcium levels. The patient was treated with standard therapy for hypercalcaemia, including aggressive intravenous hydration, bisphosphonates, calcitonin, and corticosteroids - the latter due to the consideration of granulomatous disease (such as TB) as a possible contributing cause of hypercalcaemia. Despite these interventions, a marked clinical and biochemical improvement was only observed after discontinuation of milk intake, underscoring the significant role of dietary calcium and alkali sources in the pathogenesis and persistence of MAS.

There has been a growing call among some researchers to adopt the term calcium-alkali syndrome to better reflect the underlying mechanisms of the modern presentation, which differ notably from the original condition first linked to Sippy’s ulcer treatment. Unlike the classical version - predominantly affecting middle-aged men - the contemporary form more frequently affects women, with an average onset around the age of 50. Today, the syndrome is most often triggered by the excessive use of calcium carbonate supplements, commonly available over the counter, rather than by milk consumption. A comparable case involved a 47-year-old female patient who developed hypercalcaemia and kidney stones attributed only to calcium supplementation [6].

Currently, MAS is responsible for more than 10% of all hypercalcaemia cases and stands as the third leading cause of elevated calcium levels among hospitalised individuals, surpassed only by hyperparathyroidism and cancer-related aetiologies.

Interestingly, our case does not fully align with either the classical or modern descriptions of MAS. Although the patient had no significant kidney injury and supplementation with calcium and vitamin D had already been stopped, the hypercalcaemia persisted until milk intake was discontinued. This suggests a presentation that literally reflects the historical name - “milk-alkali syndrome” - with both hypercalcaemia and metabolic alkalosis directly attributable to milk ingestion. Such a presentation reinforces the relevance of this historical terminology, even in modern clinical settings where the aetiology and patient demographics have otherwise evolved.

This case highlights the critical importance of obtaining a thorough dietary history when evaluating patients with hypercalcaemia, as excessive intake of calcium-rich foods such as milk can be a key contributing factor. MAS, though less common today, remains a relevant diagnosis in patients consuming large amounts of calcium and absorbable alkali. Management is largely supportive and centres on prompt cessation of the offending agent - in this case, excessive milk intake. While most patients experience clinical and biochemical improvement within 24 to 48 hours, serum calcium levels may remain elevated for days to weeks, and in some cases, months. Bisphosphonates have been used in select cases, but current evidence does not support their routine use in MAS. Clinicians should also monitor for transient hypocalcaemia during the recovery phase, which may require temporary calcium supplementation. This case underscores the importance of considering diet as a significant and modifiable factor in the development and management of hypercalcaemia [7-9]. 

Taking into consideration that hypercalcaemia can also be a manifestation of pulmonary TB, though this is rare. In such cases, the hypercalcaemia is attributed to excessive extra-renal 1-alpha hydroxylase activity by activated macrophages within granulomas, which leads to increased production of 1,25-dihydroxyvitamin D and enhanced intestinal calcium absorption [10,11]. Accordingly, one of the primary interventions in TB-associated hypercalcaemia is the discontinuation of oral calcium and vitamin D supplementation [10]. Although corticosteroids have been used to suppress calcitriol production in granulomatous diseases like TB, their use remains controversial due to potential immunosuppression and the risk of exacerbating the underlying infection. Our patient, diagnosed with pulmonary TB and suspected macrophage activation syndrome, was treated with corticosteroids in view of possible granulomatous-induced hypercalcaemia. However, the absence of a sustained response until milk intake was discontinued suggests that excessive dietary calcium, rather than granulomatous disease, was the dominant contributor in this case. This further emphasises the pathophysiological overlap and the importance of considering multiple concurrent causes of hypercalcaemia in complex presentations [12,13].

Limitations

Several limitations in this case warrant discussion. First, although acute kidney injury (AKI) is traditionally considered a key feature of MAS, our patient did not exhibit any renal impairment. While AKI is commonly observed, largely due to hypercalcaemia-induced vasoconstriction and impaired renal function, it is not universally present. Recent literature emphasises that the core diagnostic elements of MAS remain hypercalcaemia, metabolic alkalosis, and the ingestion of absorbable alkali, even in the absence of renal dysfunction. This reinforces the need to consider MAS in the differential diagnosis of hypercalcaemia when these criteria are met.

Second, the patient had been on regular calcium and vitamin D supplementation, which had been discontinued one week prior to the peak in serum calcium levels. While the supplements may have contributed to calcium loading, the delayed normalisation of serum calcium, only occurring five to six weeks after discontinuation, suggests that excessive milk intake was the primary driver of the persistent hypercalcaemia. Notably, biochemical improvement was only observed after milk intake was ceased, further implicating it as the main offending agent. This case highlights the need for a comprehensive literature review on the long-term effects of calcium supplementation, including how delayed or cumulative excessive intake may contribute to hypercalcaemia, particularly in individuals with other sources of calcium or alkali.

Additionally, although PTHrP was not done, a relevant investigation for malignancy-associated hypercalcaemia, extensive imaging, including MRI of the cervical, thoracic, lumbar, and sacral spine, was conducted due to the patient’s background of TB. These scans did not reveal any osteolytic lesions or suspicious masses, making a malignancy-driven process less likely. Further tests, such as serum and urine protein electrophoresis or immunofixation, could have been considered to rule out conditions such as multiple myeloma. In addition, TSH, cortisol, and 24-hour urine calcium assessments should have been performed to further narrow the differential diagnosis. Given the imaging results and overall clinical context, malignancy was considered unlikely. Together, these limitations underscore the complexity of diagnosing MAS and highlight the importance of integrating clinical, dietary, biochemical, and imaging findings when evaluating hypercalcaemia.

## Conclusions

This case underscores the importance of considering MAS as a potential and reversible cause of hypercalcaemia, particularly in patients with atypical presentations and coexisting conditions such as TB. Despite extensive investigations and standard hypercalcaemia management, including cessation of calcium/vitamin D supplements, intravenous fluids, bisphosphonates, calcitonin, and corticosteroids, the patient’s calcium levels remained persistently elevated until his excessive milk intake was identified and stopped. The resolution of symptoms and normalisation of serum calcium following dairy cessation strongly implicated milk ingestion as the primary driver. This highlights the critical role of a thorough dietary history in cases of hypercalcaemia and illustrates that MAS can present even in the absence of classic features such as renal impairment. Clinicians should remain vigilant for this often-overlooked diagnosis, particularly when evaluating hypercalcaemia in patients with complex medical backgrounds.
